# A method for mapping and quantifying whole organ diffusion-weighted image distortion in MR imaging of the prostate

**DOI:** 10.1038/s41598-017-13097-6

**Published:** 2017-10-05

**Authors:** Andrew B. Gill, Marcin Czarniecki, Ferdia A. Gallagher, Tristan Barrett

**Affiliations:** 1 Department of Radiology, University of Cambridge, Cambridge, UK; 2 Department of Medical Physics, Cambridge University Hospitals, Cambridge, UK; 3 Department of Radiology, Masovian Brodno Hospital, Warsaw, Poland; 4 Department of Radiology, Cambridge University Hospitals, Cambridge, UK; 5 CamPARI Clinic, Addenbrooke’s Hospital and University of Cambridge, Cambridge, UK

## Abstract

A computational algorithm was designed to produce a measure of DW image distortion across the prostate. This algorithm was tested and validated on virtual phantoms incorporating known degrees and distributions of distortion. A study was then carried out on DW image volumes from three sets of 10 patients who had been imaged previously. These volumes had been radiologically assessed to have, respectively, ‘no distortion’ or ‘significant distortion’ or the potential for ‘significant distortion’ due to susceptibility effects from hip prostheses. Prostate outlines were drawn on a T2-weighted (T2W) image ‘gold-standard’ volume and on an ADC image volume derived from DW images acquired over the same region. The algorithm was then applied to these outlines to quantify and map image distortion. The proposed method correctly reproduced known distortion values and distributions in virtual phantoms. It also successfully distinguished between the three groups of patients: mean distortion in ‘non-distorted’ image volumes, 1.942 ± 0.582 mm; ‘distorted’, 4.402 ± 1.098 mm; and ‘hip patients’ 8.083 ± 4.653 mm; *P* < 0.001. This work has demonstrated and validated a means of quantifying and mapping image distortion in clinical prostate MRI cases.

## Introduction

Prostate cancer is the most common non-cutaneous malignancy in men, and accounts for almost 1 in 5 of all new male cancer diagnoses^1^. Diagnosis of prostate cancer has recently undergone a paradigm shift with multi-parametric MRI (mpMRI) incorporating functional sequences being used increasingly and at earlier time points in the investigative pathway. This has placed the emphasis on both lesion detection and characterisation and necessitates high quality MR imaging.

The recently updated PI-RADS guidelines have emphasised the importance of diffusion-weighted (DWI) imaging, whilst downplaying the role of dynamic-contrast-enhanced (DCE) MRI^2^. Furthermore, recent studies assessing the use of bi-parametric MRI using DWI alone as a functional sequence underline the value of DWI^3,4^. However, despite its utility, DWI is particularly prone to artefact, with the echo planar readouts being susceptible to magnetic field in-homogeneities prevalent at the air tissue boundary of the prostate and rectum. This leads to accumulated phase errors in the readout^5–7^.

Attempts have been made to reduce artefact by employing novel sequences. Examples are the acquisition of opposite phase encoding polarities to correct magnetic field inhomogeneity^8^, reduced field of view imaging^9,10^, or correcting for B_0_ distortion effects as a post-processing step^11^. Typically studies assessing novel sequences use reader reported scoring systems; however, these are qualitative in nature and inherently subjective, being prone to inter-observer variation^12–14^. It would therefore be useful to objectively map out areas where distortion is maximal on DWI and attempt to quantify the amount of distortion present as a means of comparing sequences. Knowledge of distortion location and extent may also help in the interpretation of mpMRI and enable more accurate delineation of a lesion in the context of subsequent targeted biopsy or in cases of gland-sparing focal therapy, where complete coverage of lesions is essential.

In this study, we present a novel method for producing quantitative DWI distortion maps. First we describe the development of this model using computational ‘virtual’ phantoms, and subsequently we test the model in the clinical setting of mpMRI in groups of patients with no, or significant distortion on diffusion-weighted imaging and in a separate group of patients with hip prostheses.

## Methods

### Computational algorithm

We considered the case of two imaged organ volumes: a non-distorted ‘gold-standard’ volume and an organ volume with supposed distortion present. In the subsequent patient experiments the gold-standard volume was from a high-resolution T2-weighted acquisition; a standard DWI acquisition gave rise to the ‘distorted image volume’. Outlines were drawn around the organ to be studied all on image slices in the two volumes. In image processing terms the ‘gold-standard’ organ outlines were treated as the ‘fixed volume’ and the ‘distorted’ organ outlines as the ‘moving’ volume.

**Figure 1:**
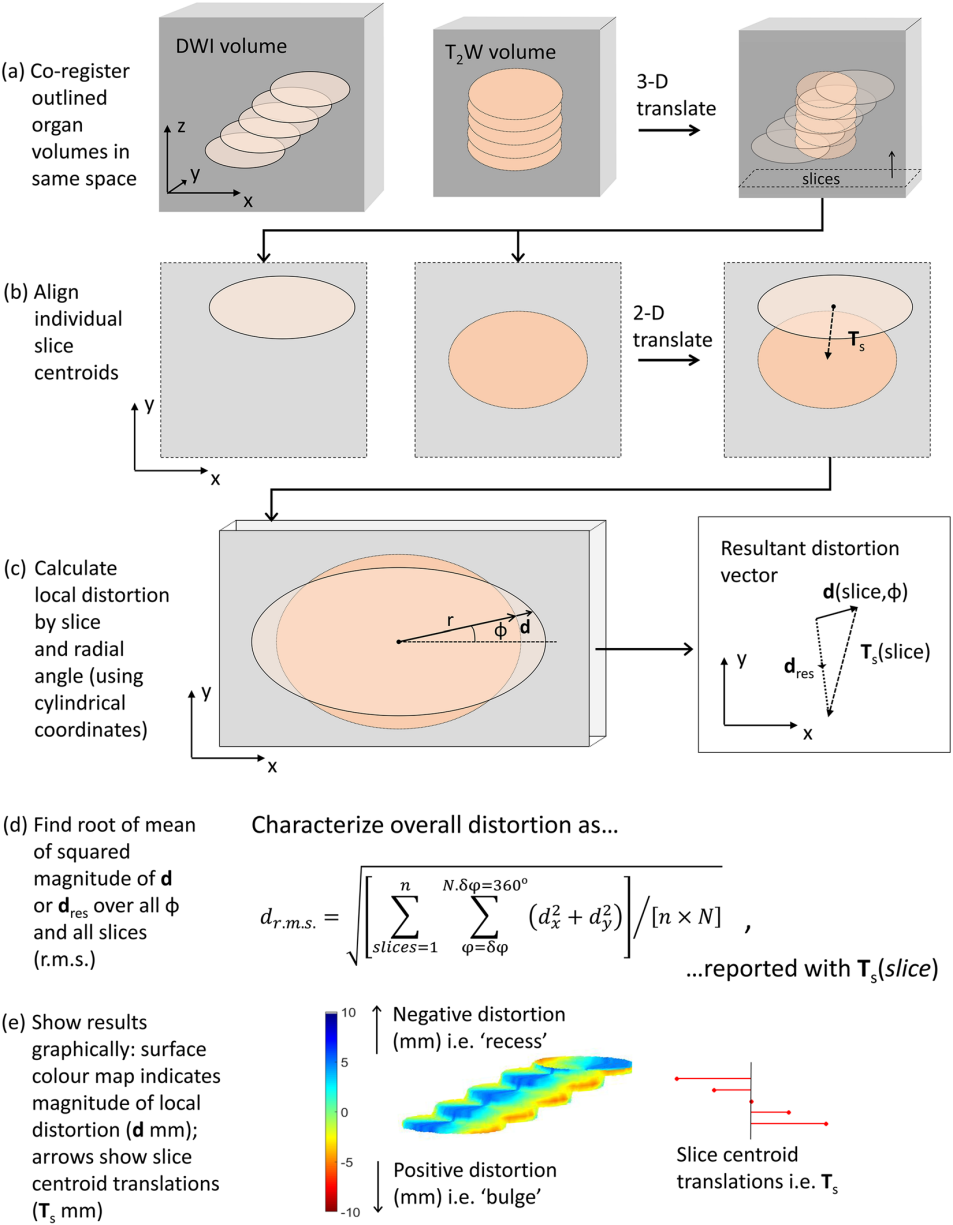
Algorithm to quantify image volume distortion. (**a**) Co-register via a 3-D translation transform the two volumes to be assessed (e.g. DWI and T2W) leaving the resulting volumes interpolated to the same coordinate space. (**b**) For each slice, align volume sections so that their centroids are coincident recording the translation **T**_s_(*slice*) necessary to achieve this. (**c**) Calculate the local radial distortion vector **d**(*slice*, *ϕ*) at the surface of each volume section using cylindrical polar coordinates. Also calculate the resultant distortion vector **d**_res_(*slice*, *ϕ*) = **d**(*slice*, *ϕ*) + **T**_s_(*slice*). (**d**) Express total distortion at surface as a root-mean-square value d_rms_ for both **d** and **d**_res_. (**e**) Show the results graphically as a colour-coded 3-D surface distortion map together with a ‘tree’ of slice centroid translations.

The algorithm to quantify organ-surface distortion was split into several steps (Fig. 1). The first step was to spatially co-register the ‘moving’ volume to the ‘fixed’ volume using a rigid translational transform. The result was expressed as a volume interpolated into the space of the fixed volume. This first step achieved two objectives: firstly, any gross translational effects due to, for example, patient or bed motion were nullified. Secondly, any difference in acquisition matrix, slice thickness, and pixel size was corrected by the spatial interpolation.

There then followed an assumption about the remaining organ-surface distortion, namely that this could be resolved broadly into two components: a rigid translation within the slice plane and a local deformable boundary distortion. The former was expressed as a translation between the centroids of the DWI and T2W outlines on the slice in question; the latter could readily be explored using a cylindrical polar co-ordinate system. The reason for considering the total distortion in these two components was to accommodate the prevailing type of distortion present in these images. This could be characterised by a predominantly linear off-set in the phase-encode direction generated by the DW echo-planar imaging sequence, combined with a more general susceptibility-based shape distortion. Since also the slice thickness was in general much larger than the in-plane pixel dimension, the local distortion was best assessed in cylindrical co-ordinates rather than in spherical coordinates.

To summarise, following the 3-D volume rigid registration (translation) the centroids of the slice outlines (‘moving’ and ‘fixed’) were found in a slice by slice fashion, as assessed in the ‘fixed’ image space. The ‘moving’ slice outlines were translated so that the two centroids were coincident. The translations required to achieve this, **T**_s_(slice) were recorded.

Local distortion, **d**(*slice, ϕ*), was then assessed as the additional (*x*,*y*) radial vector from the ‘moving’ slice outline to the ‘fixed’ slice outline, evaluated at an interval δ*ϕ* in radial angle from the common centroid point. A further measure, termed the ‘resultant distortion’ **d**_**res**_(*slice, ϕ*) was formed from the vector addition of **d** and **T**_s_: this gave a measure of the combined effects of translational and local distortions.

Finally, a measure of the total mean distortion over the whole organ was formed by calculating the root-mean-square (r.m.s.) value of the magnitude of the distortion vector (**d** or **d**_res_) summed over all slices and angles *ϕ*.

The error on the r.m.s distortion measurement was expressed as the standard error on the mean distortion where the random error on the individual radial distortion measurements was estimated to be equal to the larger in-plane pixel dimension. Here we assumed that boundary sampling to nearest neighbour coordinates in pixelated images is a normally distributed random process, which should serve as a reasonable first approximation. This measure of error does not include any systematic or random errors incurred in the outlining process itself.

The results were represented graphically by mapping a colour heat map to the distorted volume surface according to the magnitude and radial sign of **d**(*slice, ϕ*). Thus local ‘recesses’ in the distorted volume, as assessed against the gold-standard, were coloured blue and ‘bulges’ were coloured red. These maps were quantitative in the sense that the colour codes represent real distances (in mm). This colour code was assessed after centroid translation. In order to give a graphical representation of the magnitude of these slice centroid translations a ‘tree’ of **T**_s_ vectors was displayed to scale (mm) in the same image.

Distortion was also be assessed by organ sub-region by forming the root-mean-square value across a sub-range of slices or radial angle *ϕ*.

This algorithm was implemented by custom software written in MATLAB (Natick, MA) (version 2016a).

### Virtual phantom test set

A ‘virtual’ phantom in this context is a synthetic MRI volume composed of standard DICOM format images, which can be used as input to an image analysis algorithm in place of a clinically derived image volume.

In order to ensure a representative test of the software, no other adjustment to the analysis code was made apart from the source of input images. The essential concept was to form input image volumes which incorporated known features in order to compare these with the software’s eventual output and hence validate the programming code used.

For instance, a simple virtual phantom for the algorithm illustrated in Fig. 1 might consist of a contiguous stack of circular regions on successive slice images and be compared with a similar stack of circles with a slightly different diameter. The r.m.s. distortion in such a case would be expected to equal the difference in radii of the two sets of circles, since the **d**(*slice, ϕ*) has this as its constant value at all angles and slices.

Similarly, the matrix (pixel dimension and slice spacing) was varied by suitable editing of the DICOM headers, allowing simulation of an imaged volume in a different reference space. An initial translation was also be applied to one virtual phantom volume (in mm): this allowed testing of stage (a) in the algorithm illustrated in Fig. 1. Local centroid translations were also simulated to test stage (b) and locally-defined known bulges and recesses in virtual phantom surfaces were used to test stages (c), (d) and (e).

Virtual phantoms of such a kind were constructed by means of customised computer code written in MATLAB. These synthetic volumes were used as inputs into the algorithm described above.

### Clinical test sets

All studies were carried out in accordance with the Declaration of Helsinki and were approved by the institutional ethics board (CUH-16-5126). The institutional ethics board waived the need for informed consent for this retrospective analysis of fully anonymized samples. All methods were performed in accordance with the relevant guidelines and regulations. In order to test the performance of the model in a clinical setting, 20 cases were selected from a previous study investigating the effect of rectal loading on MR image quality^15^. 10 cases from this study were selected where two readers scored the DWI as having “no distortion” and 10 cases selected with both readers scoring “significant mismatch to T2W imaging *or* significant warping”. Selection into each category was by evaluation of consecutive cases.

Specifically, the DW images were scored for image quality, artefact and distortion on the b-value imaging. Quality was assessed on a 5-point scale: poor = 1, suboptimal = 2, adequate = 3, above average = 4, excellent = 5. Artefact and distortion were assessed using 4-point scales; artefact: none = 1, mild, not/mildly impacting diagnosis = 2, moderately impacting diagnosis = 3, marked artefact/non-diagnostic = 4; distortion: none = 1, mild-moderate mismatch to T2W = 2, significant mismatch to T2W or mild warping = 3, significant warping = 4. “No distortion” cases were selected where both readers scored ‘1’, “distortion” cases were selected where both readers in agreement scored the cases as ‘3’ or ‘4’.

Each of these cases was also assigned a subjective assessment of rectal distension as part of this study, using a 5-point Likert scale: 1 = no stool/gas, 2 = minimal, 3 = small amount, 4 = moderate, 5 = large amount of stool/gas. In order to further test the model, 10 clinical cases were selected where patients had hip metalwork *in situ*, as this is known to induce a greater degree of susceptibility artefact on the diffusion-weighted images.

### Magnetic Resonance Imaging

MRI was performed at 3 T (MR750, GE Healthcare) using a 32 channel phased array body coil, with no endo-rectal coil. For the purposes of the study, only axial T2 and diffusion-weighted images were assessed. Axial T2 weighted fast recovery fast spin-echo parameters: TR/TE 4273/102ms, FOV 22 × 22 cm, resolution 0.8 × 0.8 mm, 1.5 signal averages. Axial Diffusion-weighted imaging (DWI) was performed using dual spin-echo planar pulse sequence: TR/TE 3775/70ms, FOV 28 × 28 cm, resolution 2.2 × 2.2 mm, with 6 signal averages; b-values of 150, 750, 1400 and 2000 s/mm^2^, with automated ADC maps. The axial T2 and DWI sequences were matched in plane and in table location, with a 3 mm thickness and 0-mm gap.

### Prostate outlining

**Figure 2:**
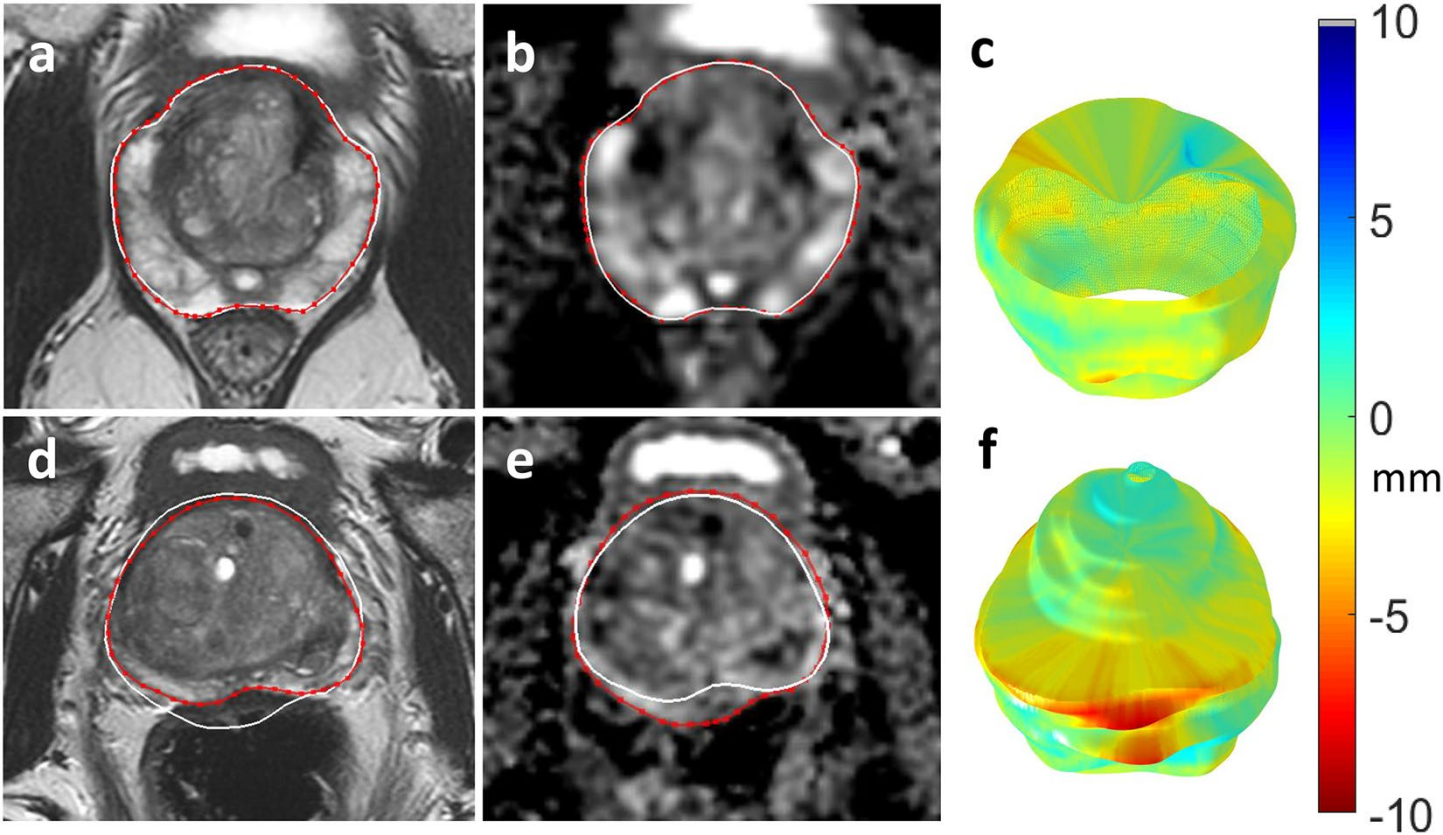
Distortion mismatch on anatomical images. 69 year-old patient on active surveillance with MRI showing no distortion on T2 (**a**) compared to diffusion-weighted imaging ADC maps (**b**); note collapsed rectum. (**d**,**e**) 68 year-old patient on active surveillance, ADC maps (**e**) show posterior distortion with warping compared to T2 imaging (**d**); note marked rectal loading. White outline of ADC map superimposed on red prostate outline on T2 image (and vice versa), to highlight the mismatch. Resulting surface distortion maps (**c**,**f**).

Whole gland (i.e. volumetric) prostate outlining was performed by a single uro-radiologist (**T.B**.) with 7 years’ experience reporting prostate MRI. Outlines were first drawn slice-by-slice on T2W axial images, in a random patient order, with the observer blinded to the clinical information. Outlines were subsequently drawn slice-by-slice on the ADC maps, using the relevant T2-axial images to ensure equivalent slices were outlined and to aid anatomical localisation (see Fig. 2). In a separate session all T2 and ADC maps were outlined a second time, using the original ROIs as reference, in order to test reproducibility.

### Statistical processing

Distortion results were examined for deviation from a Normal distribution using the Shapiro-Wilks test. Most of the result data-sets failed on this criterion meaning that non-parametric tests were used for statistical comparisons, namely the Kruskal-Wallis test and the Mann-Whitney U test.

### Data availability statement

The datasets analysed during the current study are not publicly available due to reasons of patient confidentiality.

## Results

### Virtual phantoms

**Figure 3:**
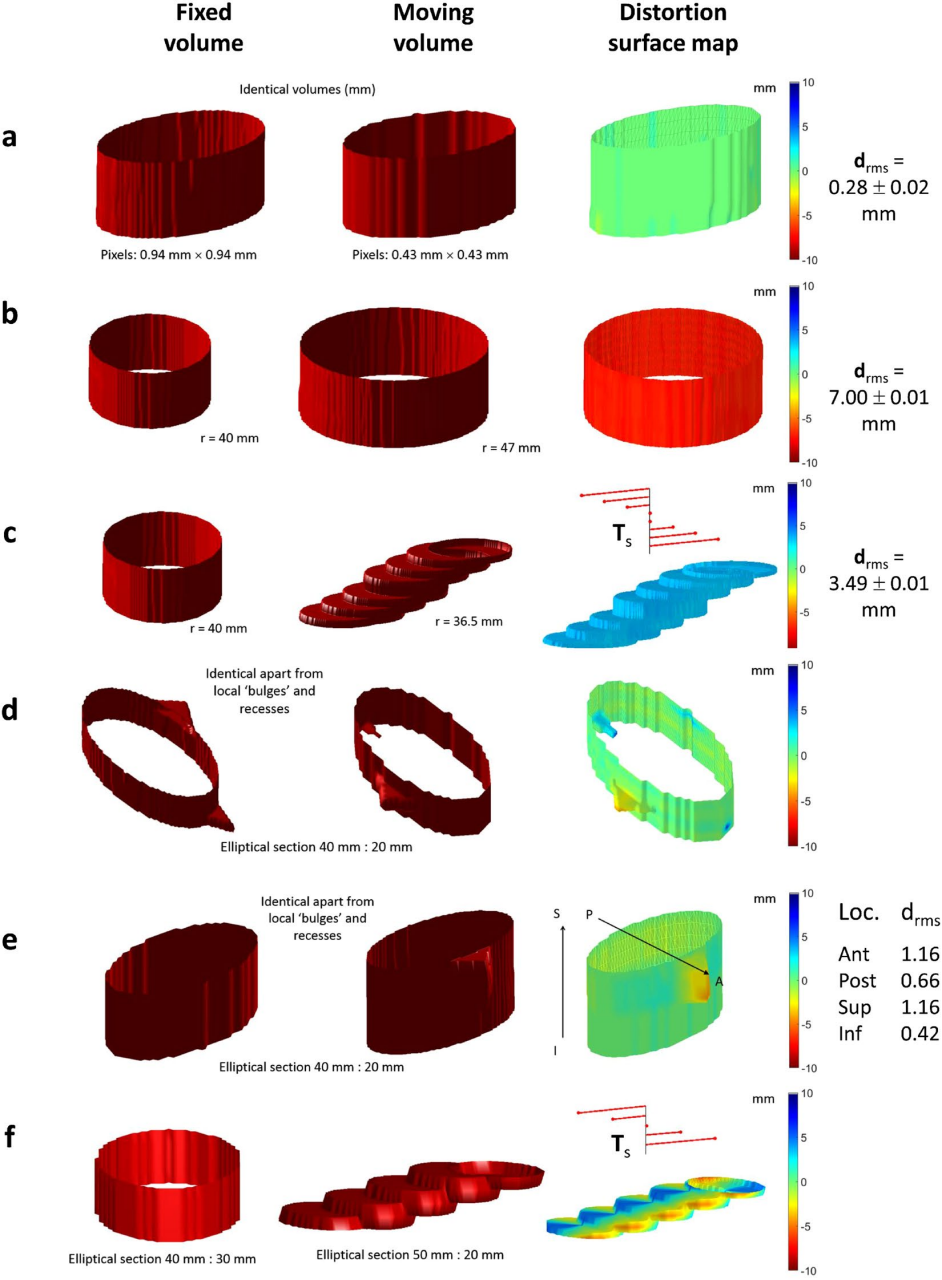
Examples of virtual phantoms used to validate the algorithm quantifying distortion. (**a**) identical volumes (as measured in mm) in dissimilar spaces; (**b**) volumes constructed from circular outlines of differing radii: **d**_rms_ reflects the difference in radii and the surface distortion map is coloured red indicating that the moving volume surface bulges from the fixed volume surface uniformly; (**c**) step-wise local translation of centroids is reflected in **T**_s_ tree while local distortion reflects the uniform recess due to circular sections in moving volume being smaller than those in fixed volume; (**d**) local bulges in otherwise identical volumes are reflected in colour of distortion surface map; (**e**) similar to (**d**) but showing an anterior and superior bias in distortion; (**f**) local translation and slice section difference in shape reflected in distortion surface map and **T**_s_ tree respectively.

A summary of the results from a variety of virtual phantoms used as input to the distortion quantification algorithm is shown in Fig. 3. Identical phantoms drawn on a different grid showed a small resultant distortion value (d_rms_ = 0.28 ± 0.02 mm), lower than the smallest pixel dimension in both images. The remaining small systematic error is probably due to interpolation of the original outlines to their respective Cartesian grids before the algorithm was applied.

Cylindrical phantoms differing only by 7 mm in radial size gave rise to d_rms_ = 7.00 ± 0.01 mm, again as expected. A stepped phantom of circular slices (radius 36.5 mm) compared against a cylindrical phantom of radius 40.0 mm gave rise to d_rms_ = 3.49 ± 0.01 mm, again close to the 3.50 mm expected. Identical phantoms drawn with local relief on the surface confirmed that the orientation of the distortion colour map was correct. Finally, a composite stepped phantom with different elliptical dimensions to an elliptical cylindrical phantom yielded a distortion map with qualitatively correct colours.

### Control experiments

**Figure 4:**
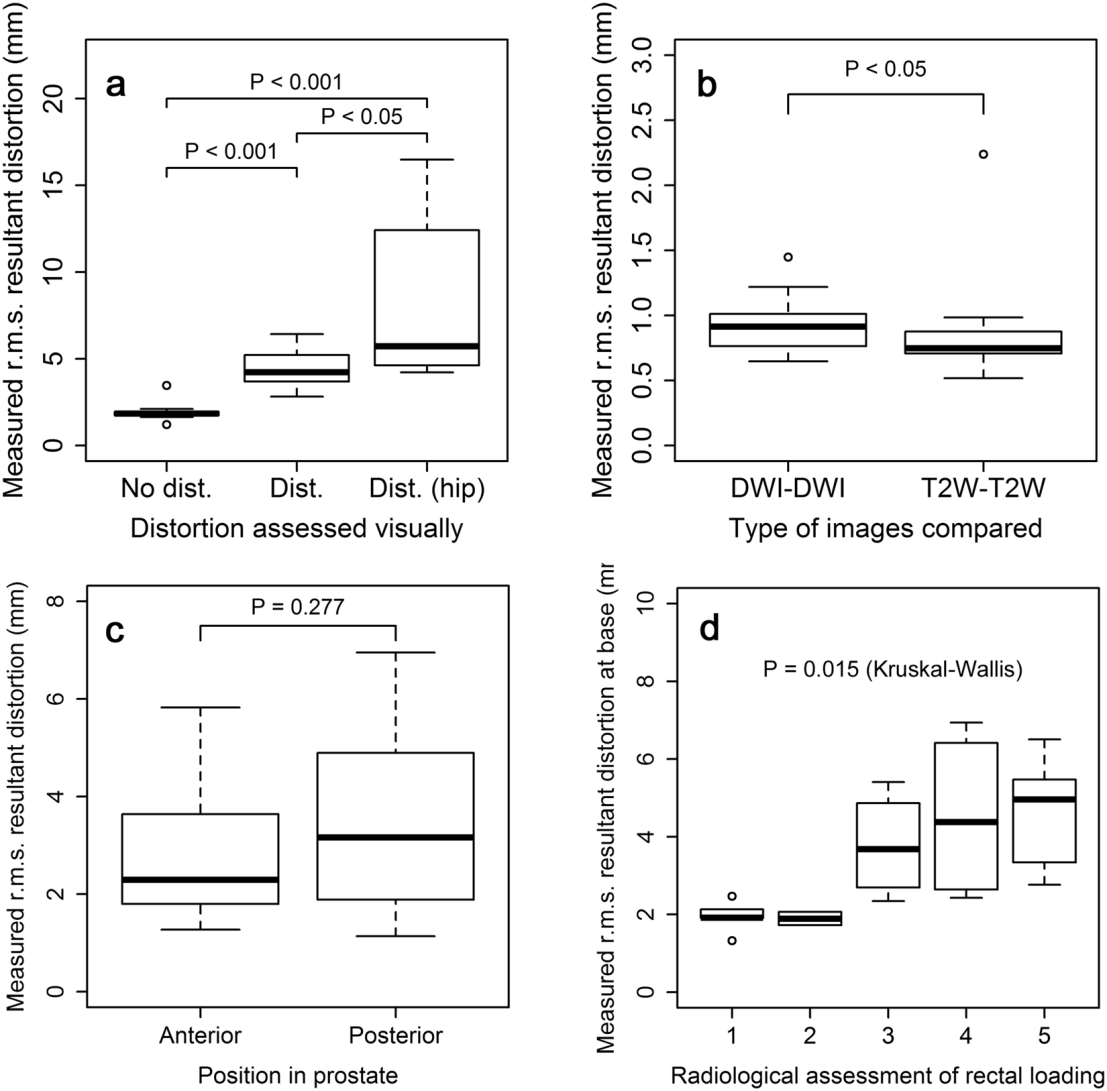
Box plots of measured values of resultant distortion (mm). (**a**) between three groups of patients: ‘distorted DWI’ and ‘non-distorted DWI’ as assessed by a radiologist, and a third group of patients with hip prostheses, most showing evidence of ‘severe warping’ in DWI; (**b**) within the control groups testing intra-observer repeatability (for both DWI and T2W images); (**c**) on DWI images by position in prostate (anterior vs posterior); and (**d**) on DWI images at base of prostate by degree of rectal loading as assessed by a radiologist on a Likert scale of 1 (none) to 5 (significant rectal loading).

The two control experiments (see Fig. 4b) whereby the outlining was repeated by the same observer on both DWI and T2W images, yielded mean (± standard deviation across the sample) r.m.s. distortion values of 0.770 ± 0.185 mm and 0.660 ± 0.299 mm respectively. The r.m.s. resultant distortion values were similar (0.927 ± 0.206 mm and 0.839 ± 0.348 mm) (Table 1). These values can be seen to be of the order of the pixel dimension on each type of image (2.2 mm and 0.8 mm). The T2W-T2W distortion values were significantly lower than those of the DWI-DWI comparison, perhaps reflecting the higher in-plane resolution of the images (*P* = 0.025).

**Table 1:** R.m.s. resultant distortion values, d_rms_ (mm), for control experiments assessing intra-observer reproducibility of outlining.

Distortion (mm)	Control images outlined			
	T2W-T2W		DWI-DWI	
Study	d_rms_	s.e.m.	d_rms_	s.e.m.
1	0.984	0.005	1.447	0.014
2	0.765	0.007	1.065	0.017
3	0.845	0.005	0.984	0.014
4	0.905	0.006	0.749	0.016
5	0.933	0.007	0.871	0.017
6	0.732	0.007	0.932	0.017
7	0.773	0.006	0.984	0.016
8	0.673	0.006	0.999	0.015
9	0.715	0.005	0.882	0.014
10	0.722	0.005	1.022	0.014
11	0.771	0.005	0.986	0.014
12	2.239	0.007	1.219	0.021
13	0.948	0.007	1.213	0.018
14	0.718	0.006	0.898	0.015
15	0.789	0.007	0.778	0.015
16	0.699	0.005	0.799	0.013
17	0.698	0.005	0.646	0.014
18	0.650	0.006	0.682	0.014
19	0.715	0.006	0.661	0.015
20	0.518	0.006	0.726	0.015
mean (sd)	0.839 (0.348)		0.927 (0.206)	

Prostate organ outlines were drawn on T2W images twice by the same observer and distortion assessed between these sets of outlines. Similarly, outlining was repeated on the set of DW images. Error on d_rms_ is given as the standard error on the mean; sample means are stated with standard deviation across the sample.

### Non-distorted and distorted DWI

The DW image quality was scored as at least adequate (≥3/5) by both readers in all 10 cases with “no distortion” (mean score 4.5 for reader 1 and 3.8 for reader 2). For “distortion” cases, reader 1 scored only 5/10 cases as adequate (score “3”) and reader 2 scored 4/10 as adequate; the remaining cases were either of poor or suboptimal quality (mean scores for reader 1 and 2 were 2.5 and 2.3, respectively). Full results for image quality, distortion and artefact are listed in Supplementary Table S2.

**Figure 5:**
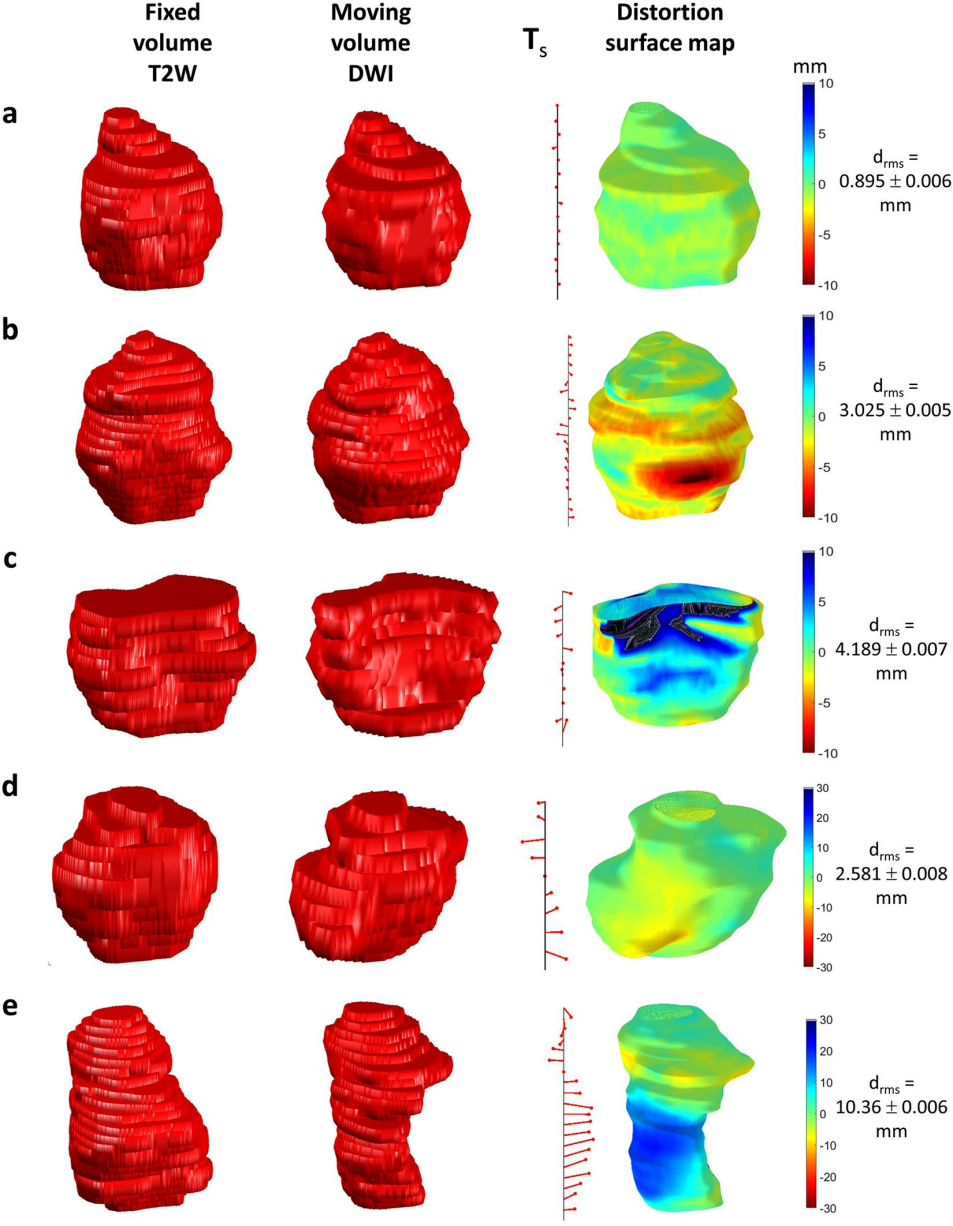
Sample distortion maps for DWI of patients referenced to T2-weighted imaging. (**a**) An example of little apparent distortion; (**b**) positive distortion i.e. ‘bulges’ in DWI volume; (**c**) negative distortion i.e. recesses in DWI volume; (**d**) patient with hip replacement, but comparatively little DWI distortion (however, note change in colour scale); (**e**) as (**d**) but showing large distortions caused by susceptibility effects. See Supplementary Video S1 for multi-orientation views of the distortion surface map shown in (**d**).

Images showing distortion colour maps are shown in Fig. 5a,b and c for a sub-set of the 20 cases of DW images radiologically assessed as either ‘distorted’ or ‘non-distorted’. The computational algorithm gave the r.m.s. resultant distortion results illustrated in Fig. 4a (mean non-distorted, 1.943 ± 0.582 mm; and distorted, 4.402 ± 1.098 mm) (Table 2). The difference in computed distortion between the two groups was statistically significant (*P* = *4.33e-05*). Mean posterior distortion was greater than anterior distortion assessed on this patient group although the difference was not statistically significant (*P* = 0.277) (Fig. 4c). Distortion at the prostate base showed an increasing trend with increasing radiologically-assessed rectal loading: the differences in distortion assessed against rectal load were statistically significant (Kruskal-Wallis, *P* = 0.015) (Fig. 4d).

**Table 2:** R.m.s. resultant distortion values, d_rms_ (mm), for three categories of patient: prostate DW image volumes radiologically assessed as having ‘no distortion’ or ‘some distortion’ or patients with hip prostheses incurring the possibility of ‘severe image warping’ due to susceptibility effects.

Distortion (mm)								
No distortion			Distortion			Distortion (hip patients)		
Study	d_rms_	s.e.m.	Study	d_rms_	s.e.m.	Study	d_rms_	s.e.m.
1	3.456	0.005	11	5.489	0.005	21	6.792	0.009
2	1.936	0.007	12	5.214	0.007	22	12.419	0.008
3	1.864	0.005	13	3.684	0.007	23	8.624	0.008
4	1.635	0.006	14	6.415	0.006	24	4.220	0.009
5	2.097	0.007	15	4.449	0.007	25	4.645	0.006
6	1.821	0.007	16	4.015	0.005	26	14.213	0.006
7	1.926	0.006	17	3.693	0.005	27	16.489	0.009
8	1.206	0.006	18	2.810	0.005	28	4.612	0.009
9	1.750	0.005	19	4.869	0.006	29	4.615	0.008
10	1.734	0.006	20	3.388	0.006	30	4.207	0.006
mean (sd)	1.943 (0.582)		mean (sd)	4.402 (1.098)		mean (sd)	8.083 (4.653)	

As in Table 1, the errors are expressed as standard error on the mean and sample standard deviation accordingly.

### Patients with hip prostheses

Images of DWI distortion maps are shown in Fig. 5 for two example cases of patients with hip prostheses, one displaying severe warping due to susceptibility effects. Mean r.m.s. resultant distortion for these cases was 8.803 ± 4.653 mm (Table 2) and significantly greater than the ‘distorted’ group in patients without prostheses (*P* = 0.035). Comparing all three categories of distortion shown in Fig. 4a, the difference between groups was highly significant (Kruskal-Wallis, *P* = 3.107e-05).

## Discussion

This is a study that attempts to objectively quantify and map out the location of distortion present on diffusion-weighted images of the prostate. Following proof of principle virtual phantom work, the method was validated in clinical mpMRI cases with or without significant distortion.

For the clinical data set, the T2-weighted images were used as the reference gold standard as this mirrors clinical reporting and the T2 sequence is more typically used for outlining and biopsy guidance when MRI-US fused targeted biopsy of a lesion is performed^16–18^. Furthermore, T2 images are less prone to susceptibility artefact as the fast spin echo sequence uses refocusing pulses between *k*-space sampling, thereby allowing protons to re-phase and avoiding phase error accumulation in the readout. We demonstrated that there was more distortion of the prostate posteriorly compared to the anterior location although this difference was not statistically significant. In addition there was a correlation between increasing DWI distortion at the base with increasing rectal loading, which is a validation of similar clinical findings^15^. Both these findings are expected as susceptibility artefact is commonly encountered at the interface of two substances with different magnetic susceptibilities, in this case the prostate and air within the rectum. In addition the model was validated in the clinical setting of hip metalwork which is known to induce significant artefact including distortion and warping.

**Figure 6:**
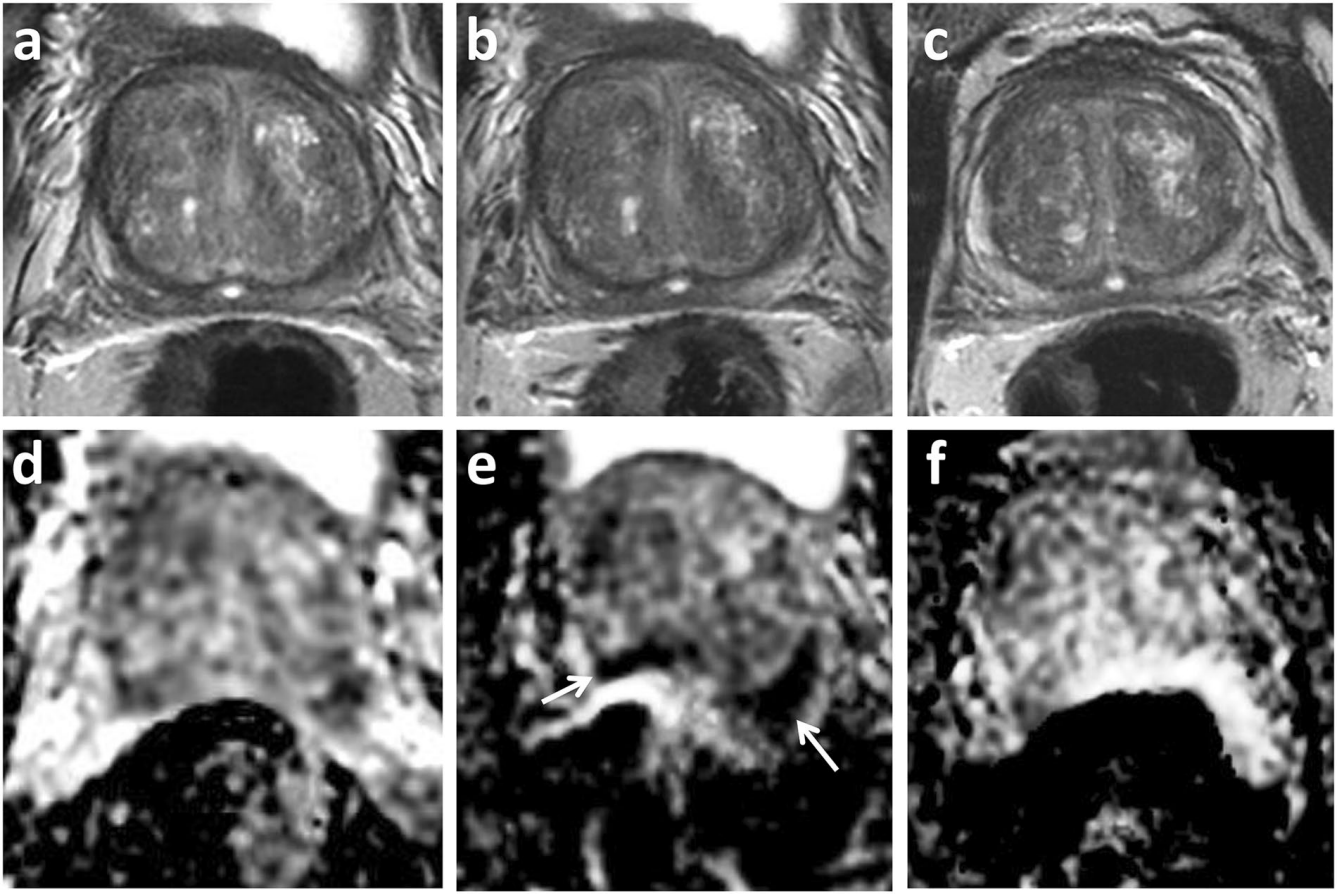
Distortion artefact in active surveillance. 66 year old patient on active surveillance. PSA 6.02 ng/ml at enrolment MRI 2014 (**a**,**d**), and stable at follow-up imaging in 2015 (**b**,**e**) and 2016 (**c**,**f**). Top row T2-weighted imaging, bottom row ADC maps. 2015 study included within current cohort shows posterior warping with apparent new and marked restricted diffusion (arrow), this is artefactual and not present on subsequent follow-up imaging.

The ability to map and quantify distortion on DWI is beneficial for assessment of novel techniques or post-processing steps aimed at reducing the degree of distortion. This will also be relevant for metal artefact reduction sequences in musculoskeletal imaging, intending to reduce in-plane artefacts and through-plane artefacts due to metal in an adjacent plane^19^. Knowledge of location and extent of distortion may additionally be helpful in a number of clinical settings. MRI is increasingly being used in biopsy naïve patients in order to identify lesions and target subsequent biopsy. DWI is seen as the key sequence for interpretation of the peripheral zone, where the majority of tumours are located, and is the secondary sequence for the transition zone. Although minor distortion may not affect the targeting of larger lesions^20^, knowledge of distortion will be necessary when outlining smaller targets. Focal therapy for prostate cancer is emerging as a management strategy for intermediate risk prostate cancer^21^. Herein, planning is often performed on pre-operative diagnostic MRI and accurate lesion delineation is essential to ensure adequate treatment coverage and a successful outcome^22^. MRI is increasingly being used in active surveillance protocols and imaging features suggesting lesion progression are used as either a trigger for repeat biopsy, or a switch to active therapy^23^. Distortion artefact in this scenario may mistakenly be assigned as MRI progression (Fig. 6) and could therefore lead to unnecessary intervention.

From a computational point of view, the algorithm assessing radial distortion can fail or partially fail in the case of highly concave local boundaries within a particular slice. Such boundary shapes are sometimes observed in prostate outlining although usually only at the base slice; here, partial volumes formed as a result of the relatively large slice thickness probably give rise to a greater error in recorded distortion. Additionally, in some contrived situations the initial 3-D translation co-registering the two volumes could lead to a severe mismatch of slices in the slice–direction (*z*). This might cause the subsequent slice by slice calculation of distortion to fail. In the context of the prostate this seems to be observed only in the most highly distorted volumes and usually affects just one peripheral slice. It will also be apparent that algorithm uses only organ surface information: no account is made of internal structure or local internal distortion within the organ. Finally, the question as to whether the r.m.s. distortion or the r.m.s. resultant distortion gives a ‘better’ measure of total distortion is largely left for further investigation although it is probable that the former metric would be more useful in volumes with little distortion, the latter when distortion is more marked.

Our study has some limitations. There is known to be both inter-and intra-observer variability in MRI-based prostate segmentation^24–26^, however, we only assessed intra-observer effects with the reproducibility data. The findings may therefore be user specific, i.e. necessitating an experienced radiologist, and the same observer performing all delineations of the prostate. T2 was used as an anatomical reference to outline the prostate ADC maps, due to the limited spatial and contrast resolution on the latter. Although the observer was blinded to clinical history, this has the potential to introduce bias as distortion will be apparent to an experienced reader, however, this situation is reflective of real-life clinical practice. We did not look at the question of incremental value of distortion maps for lesion detection, but this could form the basis for future work.

The distortion-measuring technique we have presented here could potentially be used to set a benchmark for diagnostic vs. non-diagnostic DW imaging and, as such, would be useful in the evaluation of new DW techniques. It could additionally be applied to compare anatomical imaging with other functional sequences, such as clinically used dynamic contrast-enhanced (DCE) MRI, or experimental sequences such as diffusion kurtosis imaging or diffusion tensor imaging.

In summary, we have presented a novel method for producing quantitative DWI distortion maps in order to demonstrate areas of mismatch to anatomical T2 imaging, which has a number of potential applications in both clinical and research settings.

## Electronic supplementary material

Supplementary Video S1

Supplementary Table S2
