# Supplementary figures and images for: A method for mapping and quantifying whole organ diffusion-weighted image distortion in MR imaging of the prostate
Source: Sci Rep. 2017 Oct 5;7:12727. doi: 10.1038/s41598-017-13097-6 (PMC5629196; doi:10.1038/s41598-017-13097-6)

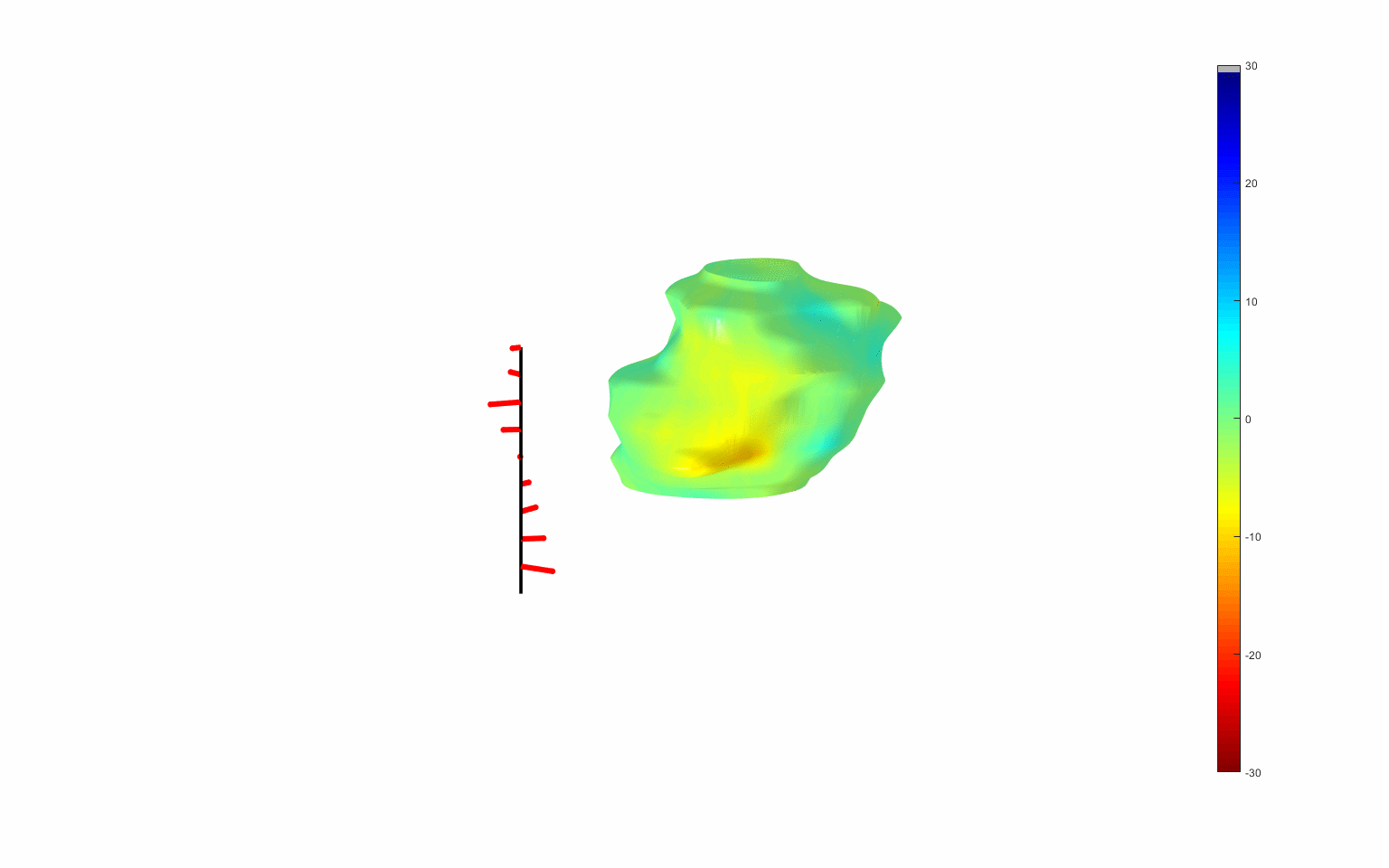

Supplement: Supplementary file 1 — Supplementary Video S1 [file 41598_2017_13097_MOESM1_ESM.gif]
