# Supplementary material for: A method for mapping and quantifying whole organ diffusion-weighted image distortion in MR imaging of the prostate
Source: Sci Rep. 2017 Oct 5;7:12727. doi: 10.1038/s41598-017-13097-6 (PMC5629196; doi:10.1038/s41598-017-13097-6)
Supplement: Supplementary file 2 — Supplementary Table S2 [file 41598_2017_13097_MOESM2_ESM.pdf]

A method for mapping and quantifying whole organ diffusion-weighted image distortion in MR imaging of the prostate

Andrew B Gill*, Marcin Czarniecki, Ferdia A Gallagher, Tristan Barrett

Supplementary Table S2: Subjective radiologist readings for image quality, distortion and artefact (two readers)

	DWI Image Quality		DWI Distortion		DWI Artefact	
Study	Reader 1	Reader 2	Reader 1	Reader 2	Reader 1	Reader 2
**No distortion**						
1	5	4	1	1	1	1
2	3	3	1	1	2	3
3	4	3	1	1	1	2
4	4	3	1	1	2	1
5	5	4	1	1	2	1
6	5	5	1	1	1	2
7	4	4	1	1	1	2
8	5	4	1	1	1	1
9	5	4	1	1	2	2
10	5	4	1	1	2	2
**Distortion**						
11	2	2	4	4	2	2
12	2	1	4	4	1	2
13	2	2	4	4	1	1
14	2	2	4	4	1	2
15	2	2	4	4	1	2
16	3	3	3	3	2	2
17	3	3	3	3	1	1
18	3	3	3	3	1	1
19	3	2	3	3	2	2
20	3	3	3	3	1	1
**Hip replacement**						
21	2	2	4	4	4	3
22	1	1	4	4	4	4
23	2	2	4	4	4	4
24	2	2	4	4	3	3
25	3	3	4	4	3	2
26	1	1	4	4	4	4
27	1	1	4	4	4	4
28	1	2	4	4	4	3
29	2	3	4	4	3	4
30	2	2	4	4	4	3
